# Speckle-tracking echocardiography combined with imaging mass spectrometry assesses region-dependent alterations

**DOI:** 10.1038/s41598-020-60594-2

**Published:** 2020-02-27

**Authors:** Kathleen Pappritz, Jana Grune, Oliver Klein, Niklas Hegemann, Fengquan Dong, Muhammad El-Shafeey, Jie Lin, Wolfgang M. Kuebler, Ulrich Kintscher, Carsten Tschöpe, Sophie Van Linthout

**Affiliations:** 1 Berlin Institute of Health Center for Regenerative Therapies & Berlin-Brandenburg Center for Regenerative Therapies (BCRT), Charité – Universitätsmedizin Berlin, Campus Virchow Klinikum (CVK), Berlin, Germany; 2 German Center for Cardiovascular Research (DZHK), Partner site Berlin, Berlin, Germany; 3 Institute of Physiology, Charité – Universitätsmedizin Berlin, Charité Campus Mitte (CCM), Berlin, Germany; 4 Center for Cardiovascular Research (CCR), Institute of Pharmacology, Charité – Universitätsmedizin Berlin, CCM, Berlin, Germany; 5 Medical Biotechnology Research Department, Genetic Engineering and Biotechnology Research Institute (GEBRI), City of Scientific Research and Technological Applications, Alexandria, Egypt; 6 Department of Cardiology, Charité – Universitätsmedizin Berlin, CVK, Berlin, Germany

**Keywords:** Ultrasound, Cardiology

## Abstract

Left ventricular (LV) contraction is characterized by shortening and thickening of longitudinal and circumferential fibres. To date, it is poorly understood how LV deformation is altered in the pathogenesis of streptozotocin (STZ)-induced type 1 diabetes mellitus-associated diabetic cardiomyopathy and how this is associated with changes in cardiac structural composition. To gain further insights in these LV alterations, eight-week-old C57BL6/j mice were intraperitoneally injected with 50 mg/kg body weight STZ during 5 consecutive days. Six, 9, and 12 weeks (w) post injections, echocardiographic analysis was performed using a Vevo 3100 device coupled to a 30-MHz linear-frequency transducer. Speckle-tracking echocardiography (STE) demonstrated impaired global longitudinal peak strain (GLS) in STZ versus control mice at all time points. 9w STZ animals displayed an impaired global circumferential peak strain (GCS) versus 6w and 12w STZ mice. They further exhibited decreased myocardial deformation behaviour of the anterior and posterior base versus controls, which was paralleled with an elevated collagen I/III protein ratio. Additionally, hypothesis-free proteome analysis by imaging mass spectrometry (IMS) identified regional- and time-dependent changes of proteins affecting sarcomere mechanics between STZ and control mice. In conclusion, STZ-induced diabetic cardiomyopathy changes global cardiac deformation associated with alterations in cardiac sarcomere proteins.

## Introduction

Diabetic cardiomyopathy is an own clinical entity, which occurs in the absence of hypertension and coronary artery disease^1,2^. Experimental STZ-induced type 1 diabetes mellitus-associated diabetic cardiomyopathy is associated with enhanced cardiac cytokine levels and collagen I deposition, resulting in cardiac dysfunction^3^. Cumulative evidence shows that early diabetic cardiomyopathy manifests in LV diastolic dysfunction accompanied by low-grade inflammation lacking pronounced fibrosis^4^. Imaging techniques, like echocardiography^5^ facilitate detection of alterations in diastolic performance.

In general, LV architecture is composed of longitudinal and circumferential fibres, building the endo-, meso-, and epicardial layers of the heart^6^. The orientation of the fibres within those layers is comprised of a right-handed helix in the subendocardium, circumferential oriented fibres in the cardiac midwall, and a left-handed helix in the subepicardium^7^. During cardiac contraction, fibres undergo shortening and thickening, whereby contraction of the subendocardial longitudinal fibres mainly determine LV function in longitudinal direction^8^. In contrast, thickening of the mesocard contributes to circumferential and radial motion. Due to the opposite orientation of the subendocardial and subepicardial fibres, the shortening of these fibres determines cardiac twist during LV contraction. Recently, strain parameters assessed by STE, which reflect tissue deformation (strain) during the cardiac cycle, have been included in guideline recommendations for the diagnosis of heart failure^9,10^. In contrast to conventional echocardiography, STE is able to detect early subtle changes and has been shown to be of diagnostic value^11,12^. Studies in patients with heart failure with preserved ejection fraction^13^ and diabetes mellitus^14^ demonstrated that GLS was initially impaired, but subsequently compensated by circumferential or radial function to maintain ejection fraction^15^. The authors hypothesized that diabetes-associated cardiac fibrosis particularly occurs in the endo- and epicardial layers, which predominantly consist of longitudinal fibers. Therefore, enhanced fibrotic lesions in these layers will specifically cause a decline of longitudinal strain parameters^16^. To date, further evidence for this hypothesis is missing and the underlying mechanistic reason for impaired myocardial deformation during diabetic cardiomyopathy remains poorly understood^17^.

Collagen is one of the most important proteins of the extracellular matrix and ensures myocardial structure and architecture^18^. After secretion as pro-peptides, collagen molecules are cleaved, followed by stabilization via cross-linking. Matrix metalloproteinases (MMPs) are responsible for collagen degradation, indicating their role as modulators in the maintenance of tissue homeostasis as well as in pathological remodelling. In heart failure, the equilibrium between MMPs and their tissue inhibitors (TIMPs) varies depending on the pathogenic entity^19^. Since several decades it is known that an enhanced collagen deposition in diabetic patients is associated with an impairment in LV function. We recently published data, which supports the hypothesis that a decline in GLS, indicative for reduced cardiac performance, strongly correlates with enhanced collagen and TIMP-1 levels^20^. Beside the relevance of cardiac fibrosis, there is accumulating evidence that particularly dysfunctionality of the contractile apparatus^21^, involving myosin and titin^4,22^, occurs at the early stage of diabetic cardiomyopathy.

Despite enormous progress in the understanding of the diabetes mellitus-associated changes in extracellular matrix turnover and expression of contractile proteins, there is still little known about their subsequent effects on cardiac deformation behaviour. To our knowledge, this is the first study investigating impaired global deformation behaviour and associated alterations in the expression of cardiac structure components in experimental STZ-induced type 1 diabetes mellitus-associated diabetic cardiomyopathy.

## Results

### STZ-induced type 1 diabetes mellitus is associated with impaired morphological parameters

**Figure 1:**
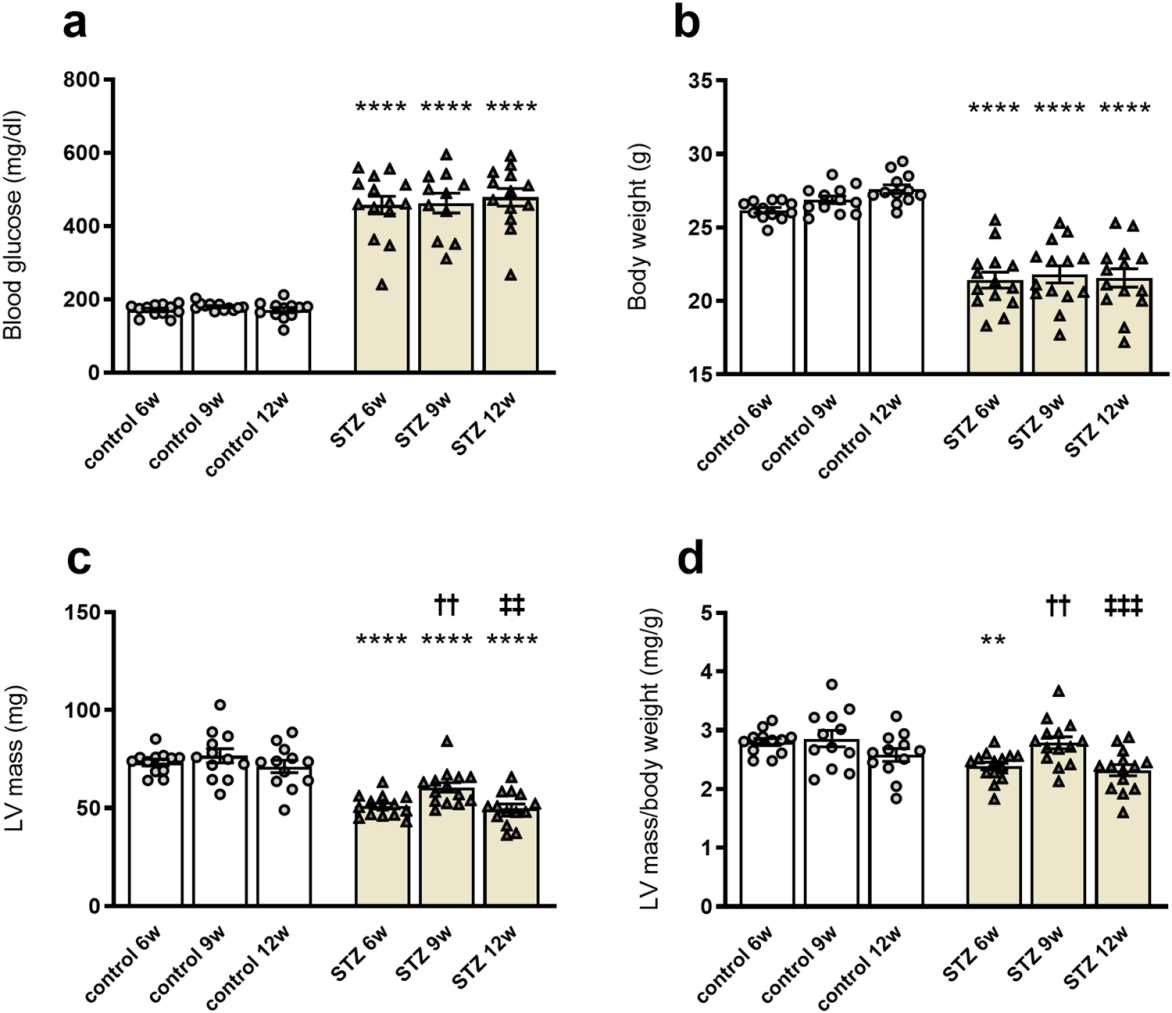
STZ-induced type 1 diabetes mellitus is associated with impaired morphological parameters. (**a**) Blood glucose levels (mg/dl) in control mice and 6w, 9w, and 12w after STZ application. Besides increased blood glucose, mice suffering from type 1 diabetes displayed lower body weight (**g**) over time (**b**). As morphological parameters, LV mass (mg; **c**) and LV mass/body weight (**d**) are depicted. Bar graphs represent the mean ± SEM. Data were analysed with One-way ANOVA or Kruskal-Wallis test (*p < 0.05; **p < 0.01, ***p < 0.001, ***p < 0.0001 versus corresponding control; ^†^p < 0.05, ^††^p < 0.01, ^†††^p < 0.001, ^††††^p < 0.0001 versus the 6w STZ; ^‡^p < 0.05, ^‡‡^p < 0.01, ^‡‡‡^p < 0.001, ^‡‡‡‡^p < 0.0001 versus the 9w STZ; n = 12/controls and n = 14/STZ).

In accordance with previous studies^23^, mice suffering from type 1 diabetes mellitus displayed increases in blood glucose levels at 6w, 9w, and 12w after STZ application versus corresponding controls, respectively (Fig. 1a). In parallel, the body weight of STZ-treated mice was lower at 6w, 9w, and 12w compared to respective control mice (Fig. 1b). Additionally, we detected a decline in the LV mass of STZ mice at all three investigated time points versus the respective controls (Fig. 1c). LV mass/body weight ratios revealed a lower LV mass/body weight ratio only at 6w after STZ application versus the corresponding control mice (Fig. 1d). Surprisingly, 9w after type 1 diabetes mellitus induction, LV mass and LV mass/body weight were higher versus 6w and 12w STZ mice, respectively (Fig. 1c,d). To further resolve the question why 9w STZ differed from 6w and 12w STZ mice, subsequent experiments and analysis were designed.

### STZ-induced type 1 diabetes mellitus impairs diastolic and systolic function

**Figure 2:**
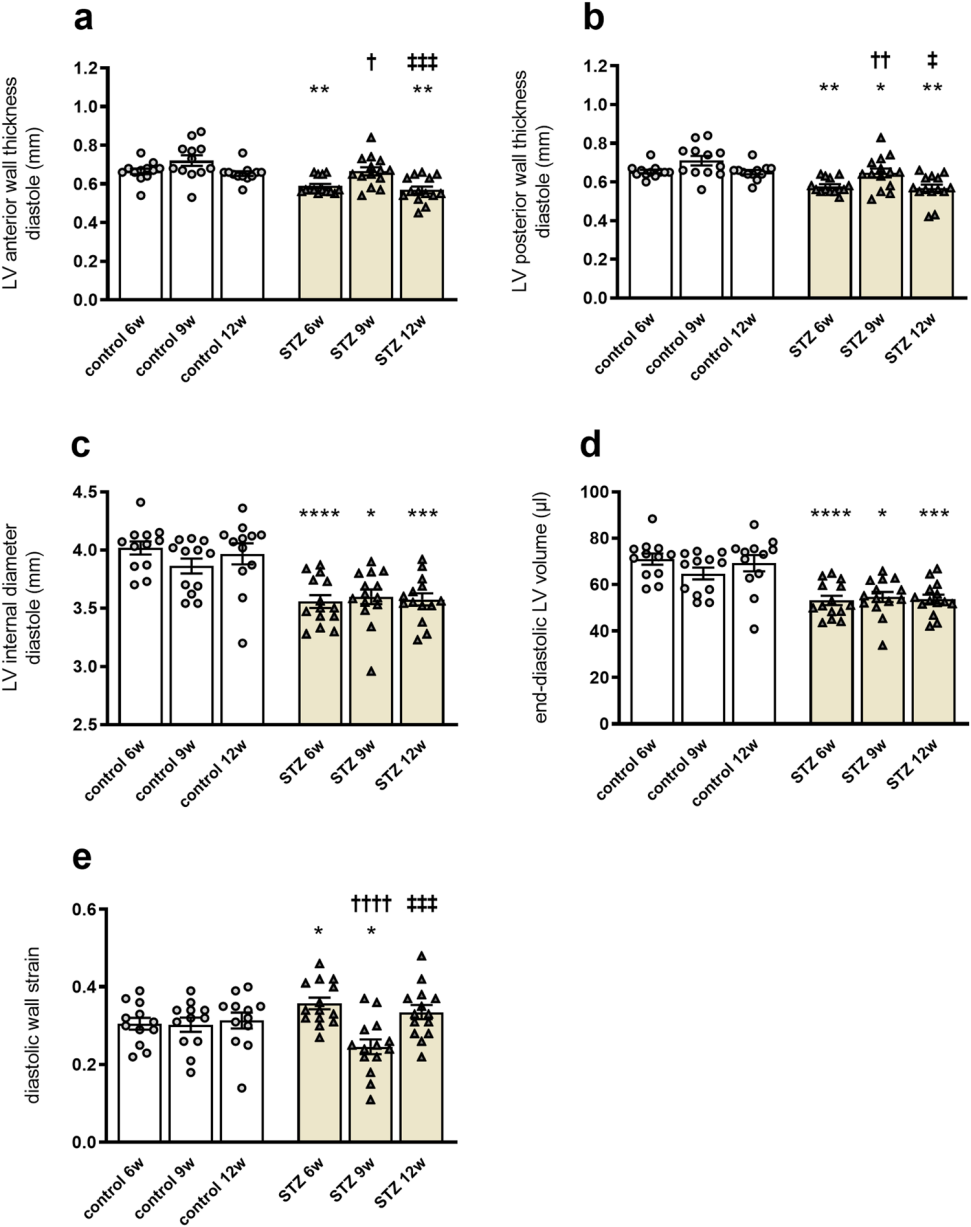
STZ-induced type 1 diabetes mellitus reduces diastolic function. To determine diastolic performance, left ventricular anterior wall (**a**), left ventricular posterior wall (**b**), LV internal diameter (**c**), and end-diastolic volume (**d**) were assessed using a Vevo 3100. In addition, diastolic wall strain (**e**) was calculated using posterior wall thicknesses during systole and diastole. Bar graphs represent the mean ± SEM. Data were analysed with One-way ANOVA or Kruskal-Wallis test (*p < 0.05; **p < 0.01, ***p < 0.001, ***p < 0.0001 versus corresponding control; ^†^p < 0.05, ^††^p < 0.01, ^†††^p < 0.001, ^††††^p < 0.0001 versus the 6w STZ; ^‡^p < 0.05, ^‡‡^p < 0.01, ^‡‡‡^p < 0.001, ^‡‡‡‡^p < 0.0001 versus the 9w STZ; n = 12/controls and n = 14/STZ).

As shown in Fig. 2, the mean diastolic LV anterior wall and LV posterior wall thickness were significantly reduced in STZ-treated mice after 6w and 12w, compared to corresponding control mice (Fig. 2a,b). Consistent with Shepherd and colleagues^24^, we detected a reduction in mean diastolic LV internal diameter in 6w, 9w, and 12w STZ mice compared to control mice (Fig. 2c). This was paralleled by a decline in end-diastolic LV volume at 6w, 9w, and 12w post-STZ versus controls (Fig. 2d). With respect to the LV mass and LV mass/body weight ratio, 9w STZ mice differed from 6w and 12w STZ mice, as indicated by increased diastolic LV anterior wall and LV posterior wall values. Specifically, LV anterior wall thickness of 9w STZ mice was higher compared to 6w STZ and 12w STZ mice, respectively. Analogously, LV posterior wall thickness was increased at STZ 9w versus STZ 6w and 12w, respectively. As reported previously^25–27^, diastolic wall strain (DWS), which displays the differences of posterior LV wall thickness during systole and diastole, is proposed to be a marker for diastolic function and correlates with LV stiffness. Compared to the respective controls, 6w STZ mice displayed increased DWS, whereas DWS was reduced in 9w STZ mice (Fig. 2e). Similar to LV wall thicknesses, differences between 9w STZ mice versus 6w STZ and 12w STZ mice were detected, respectively.

In agreement with existing data^3,24,28^, we further observed reduced cardiac ejection fraction, and fractional shortening in 9w STZ versus control mice. In addition, stroke volume and cardiac output were decreased in STZ animals compared to the corresponding control mice (Supplemental Fig. 1). Comparison within the STZ groups revealed only a reduced ejection fraction in 9w STZ mice versus 12w STZ animals.

### STZ-induced type 1 diabetes mellitus reduces global peak strain

**Figure 3:**
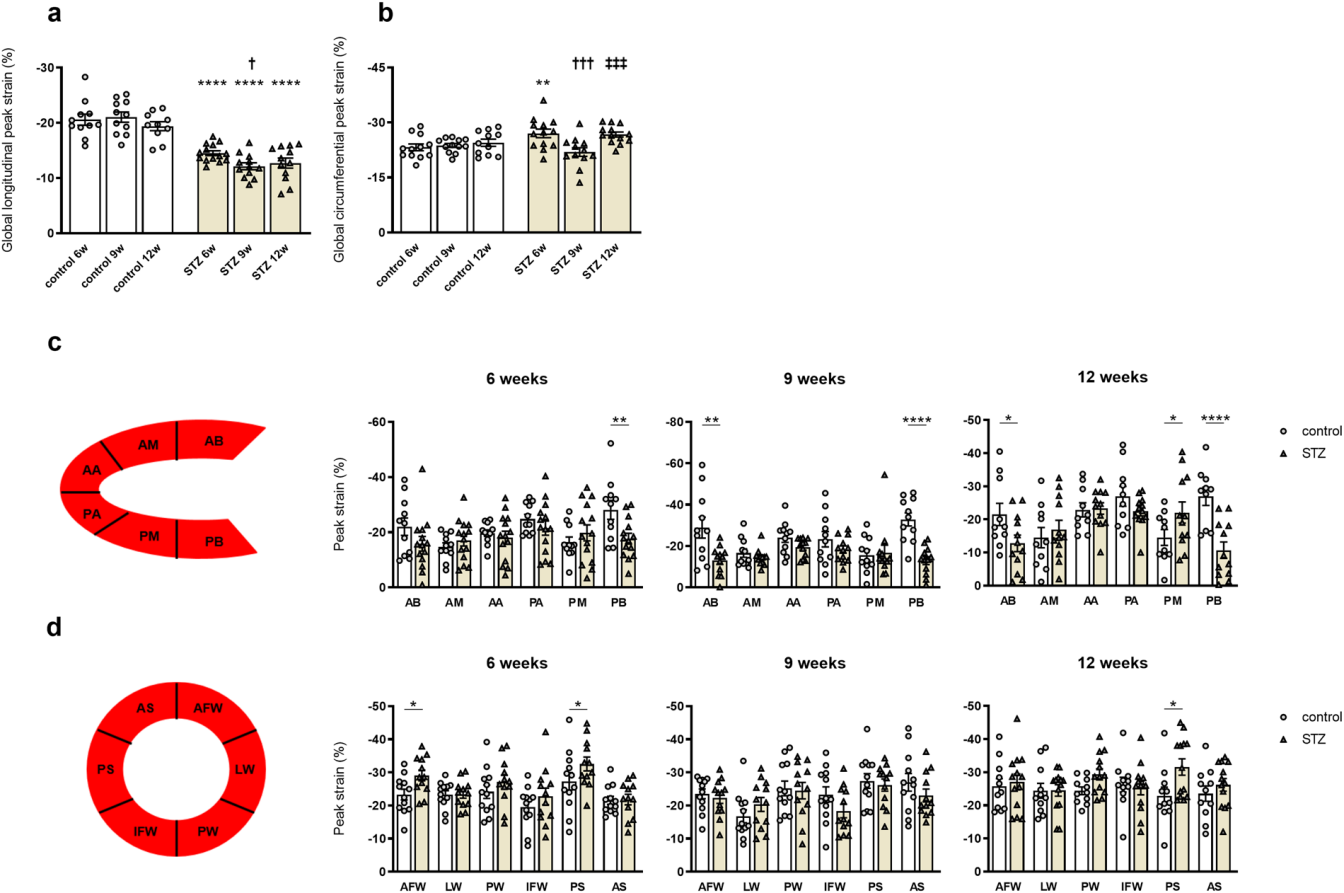
STZ-induced type 1 diabetes mellitus reduces global peak strain. Two-dimensional STE was used to further determine GLS (**a**) and GCS (**b**). Quantitative segmental analysis in long axis (**c**) and short axis view (**d**) confirmed time-dependent changes in myocardial deformation behaviour. *AA* Anterior Apex; *AB* Anterior Base; *AFW* Anterior Free Wall; *AM* Anterior Mid; *AS* Anterior Septum; *IFW* Inferior Free Wall; *LW* Lateral Wall; *PA* Posterior Apex; *PB* Posterior Base; *PM* Posterior Mid; *PS* Posterior Septal Wall; *PW* Posterior Wall. Bar graphs represent the mean ± SEM. Data were analysed with One-way ANOVA or Kruskal-Wallis test (*p < 0.05; **p < 0.01, ***p < 0.001, ***p < 0.0001 versus corresponding control; ^†^p < 0.05, ^††^p < 0.01, ^†††^p < 0.001, ^††††^p < 0.0001 versus the 6w STZ; ^‡^p < 0.05, ^‡‡^p < 0.01, ^‡‡‡^p < 0.001, ^‡‡‡‡^p < 0.0001 versus the 9w STZ; n = 12/controls and n = 14/STZ).

Next, we examined global peak strain parameters as well as segmental deformation behaviour, indicated by regional peak strain values, to evaluate myocardial function. In comparison to the corresponding controls, GLS was reduced in mice at 6w, 9w, and 12w STZ, respectively (Fig. 3a). Furthermore, the STZ 9w group displayed a decrease in GLS compared to the STZ 6w mice. Additionally performed quantitative segmental strain analysis indicated decreased myocardial deformation behaviour of the anterior and posterior base at 9w after STZ treatment (Fig. 3c). In parallel, we observed an increase in GCS in 6w STZ and 12w STZ mice versus corresponding controls, respectively (Fig. 3b). In contrast, the 9w STZ animals displayed a lower GCS relative to 6w and 12w STZ mice, respectively. Segmental analysis of the respective short axis B-mode images showed improved contraction of the anterior free wall and posterior septum in STZ mice at 6w compared to controls (Fig. 3d). Analogous, myocardial contraction of the posterior septum was increased in 12w STZ mice versus controls. These data indicate that STZ-induced diabetic cardiomyopathy is associated with an impairment in GLS, whereas GCS is partially improved. Additionally, the GLS and GCS rates, which reflect the change in strain over time, exhibited similar regulations in STZ versus control mice as the respective GLS and GCS (Supplemental Fig. 2).

### STZ-induced type 1 diabetes mellitus modulates collagen I deposition and extracellular matrix turnover in a time-dependent manner

**Figure 4:**
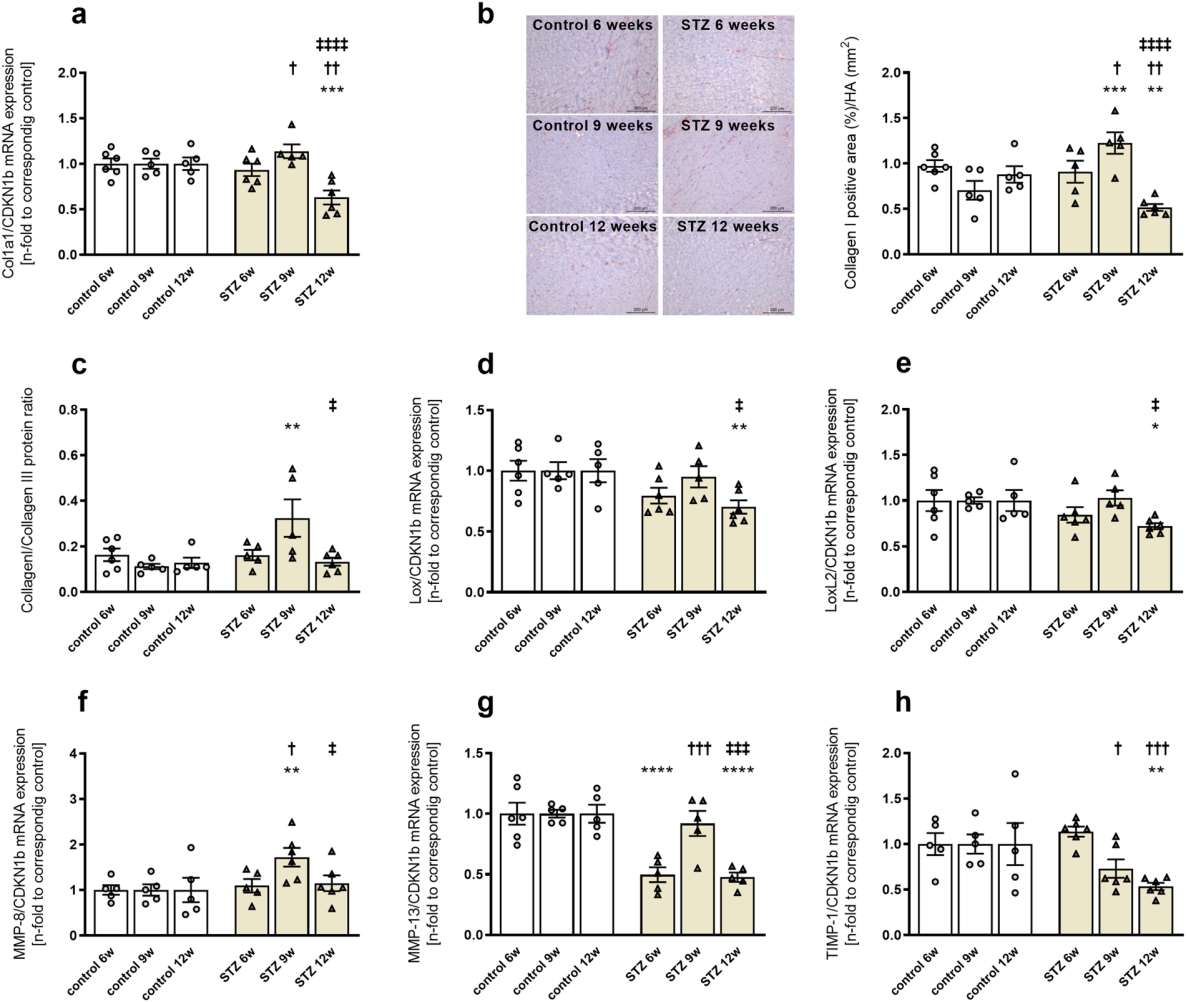
STZ-induced type 1 diabetes mellitus modulates collagen I deposition and cross-linking in a time-dependent manner. 6w, 9w, and 12w after diabetes induction, Col1a1 mRNA (**a**) and collagen I protein levels (**b**) were investigated. Immunohistological staining is shown by representative images (scale bar = 200 μm; middle panel). To further characterize cardiac fibrosis, collagen I/III protein ratio (**c**), Lox (**d**), and LoxL-2 (**e**) gene expression were measured. Additionally, mRNA levels of MMP-8, MMP-13, and TIMP-1 were detected (**f–h**). Quantification of the positive area (%)/HA (mm^2^) was performed via digital image analysis. Bar graphs represent the mean ± SEM. Data were analysed with One-way ANOVA or Kruskal-Wallis test (*p < 0.05; **p < 0.01, ***p < 0.001, ***p < 0.0001 versus corresponding control; ^†^p < 0.05, ^††^p < 0.01, ^†††^p < 0.001, ^††††^p < 0.0001 versus the 6w STZ; ^‡^p < 0.05, ^‡‡^p < 0.01, ^‡‡‡^p < 0.001, ^‡‡‡‡^p < 0.0001 versus the 9w STZ; n = 5–6/controls and n = 5–6/STZ).

After we observed time-dependent changes in STE-assessed strain parameters following STZ treatment, we investigated possible underlying mechanisms. In comparison to the respective controls, we detected no differences in Col1a1 mRNA expression in STZ 9w animals (Fig. 4a). In contrast, collagen I protein expression was increased in 9w STZ mice versus controls, which was accompanied by an increase in the collagen I/collagen III protein ratio (Fig. 4b,c). Twelve w after STZ application, Col1a1 gene expression was reduced in mice, and this finding was associated with a reduction in collagen I protein expression (Fig. 4b). In parallel to the decline in collagen mRNA and protein levels, 12w STZ mice displayed a decrease in lysyl oxidase (Lox) and lysyl oxidase-like (LoxL)-2 mRNA levels compared to controls, respectively (Fig. 4d,e). Beside collagen cross-linking, collagen homeostasis is mainly regulated by MMPs and TIMPs. Therefore, we examined the expression of MMPs and TIMPs in the LV (Fig. 4f–h). Especially, MMP-8 and MMP-13 are responsible for collagen I degradation, indicating their role in cardiac remodeling^29^. Compared to control animals, MMP-8 mRNA levels were elevated in 9w STZ mice, whereas MMP-13 mRNA levels were reduced in 6w and 12w STZ mice, respectively (Fig. 4f,g). Similar to collagen I, mice at 9w STZ displayed an increase in MMP-8 and MMP-13 gene expression compared to the 6w STZ group. Furthermore, MMP-8 and MMP-13 mRNA levels were lower in 12w STZ mice versus 9w STZ mice. TIMP-1 expression showed a decline in mRNA levels at 9w and 12w post-STZ, respectively, compared to 6w STZ (Fig. 4h).

### STZ-induced type 1 diabetes mellitus alters the cardiac proteome in a time-dependent manner

**Figure 5:**
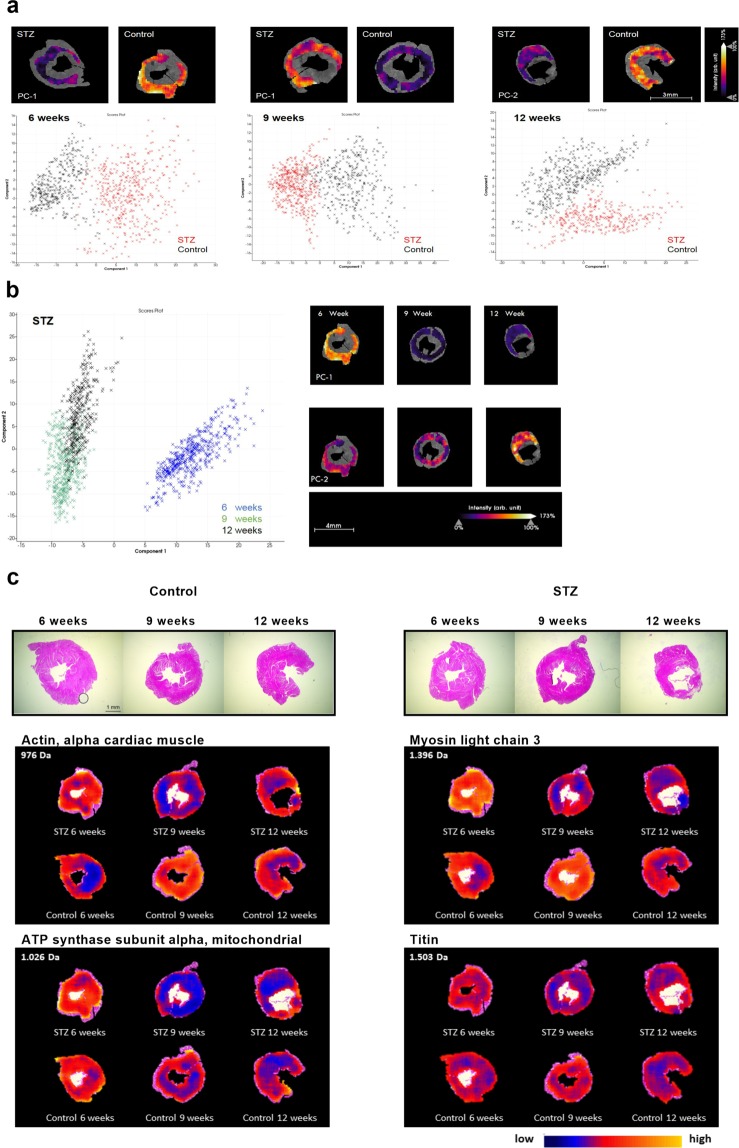
STZ-induced type 1 diabetes mellitus is associated with alterations in proteins indicative for contractile function in a region- and time-dependent manner using IMS. (**a**) Principal component analysis from LV tissues at 6w, 9w, and 12w after diabetes induction, clearly distinguished between STZ and control mice. (**b**) Principal component analysis of STZ 6w versus 9w, and 12w showed differences of in the intensity distribution within the STZ group. (**c**) H/E staining of control (left panel; scale bar = 1 mm) and STZ (right panel) mice with the corresponding spatial intensity distribution of 976 Da (cardiac α-actin), 1396 Da (myosin light chain 3), 1026 Da (mitochondrial ATP synthase subunit alpha) and 1503 Da (Titin).

To further understand the changes in strain parameters, a hypothesis-free proteome analysis was performed (Fig. 5a–c). To this end, we applied principal component analysis yields to discriminate peptide signatures between the LV tissues at the different time points. The principal component-1 clearly distinguished the protein signatures between STZ and control LV tissue at 6w, 9w and 12w (Fig. 5a). Furthermore, the comparison of the peptide signatures within the STZ group resulted in a clear distinction between STZ 6w, 9w and 12w, respectively (Fig. 5b). To further understand these changes in the cardiac proteome, the mesoderm was subsequently defined as region of interest and specific peptide values (m/z) were localized and identified (Fig. 5c). The spatial distribution of m/z values indicated a higher intensity distribution of m/z 976 Da from cardiac α-actin, m/z 1396 Da from myosin light chain 3, and m/z 1063 Da from mitochondrial ATP synthase in 6w STZ versus 6w control mice, respectively (Table 1). In contrast, the intensity distribution of α-actin, myosin light chain 3, mitochondrial ATP synthase, and titin were declined in 9w STZ mice versus corresponding controls, respectively (Table 1). Interestingly, 12w post-STZ only α-actin, myosin light chain 3, and mitochondrial ATP synthase showed a higher intensity distribution compared to the respective controls (Table 1).

**Table 1:** ROC analysis of the intensity distribution from MALDI-IMS-derived identified proteins. Peptides with lowest mass difference to the LC-MS/MS reference list value and with similar discrimination characteristics were assumed as a match.

Centroid [m/z]	±[Da]	ROC [AUC] 6w STZ vs. 6w Control	p-value 6w STZ vs. 6w Control	ROC [AUC] 9w STZ vs. 9w Control	p-value 9w STZ vs. 9w Control	ROC [AUC] 12w STZ vs. 12w Control	p-value 12w STZ vs. 12w Control	Mr IMS	LC-MS	△ [Da]	protein name
976.569	0.27	0.683099	<0.01	0.171841	<0.01	0.323569	<0.01	975.5617	975.4410	0.120691643	α-actin
1500.603	0.27	0.643767	<0.01	0.151043	<0.01	0.309509	<0.01	1499.5957	1499.7004	−0.104770521	
1956.061	0.27	0.780955	<0.01	0.277548	<0.01	0.370325	<0.01	1955.0537	1955.0363	0.017307909	
2227.932	0.27	0.807939	<0.01	0.307902	<0.01	0.314609	<0.01	2226.9247	2227.0579	−0.133232170	
2244.940	0.27	0.690501	<0.01	0.248110	<0.01	0.336561	<0.01	2243.9327	2243.0528	0.879852830	
1396.660	0.27	0.785575	<0.01	0.216586	<0.01	0.261510	<0.01	1395.6527	1395.7470	−0.094326783	myosin light chain 3
1501.683	0.27	0.752211	<0.01	0.235596	<0.01	0.352482	<0.01	1500.6757	1500.6732	0.002447283	
1722.797	0.27	0.719975	<0.01	0.377262	<0.01	0.357083	<0.01	1721.7897	1721.8406	−0.050969172	
1026.516	0.27	0.631519	<0.01	0.327338	<0.01	0.350877	<0.01	1025.5087	1025.5869	−0.078244313	mitochondrial ATP synthase
1575.657	0.27	0.678074	<0.01	0.391201	<0.01	0.407280	>0.05	1574.6497	1574.7787	−0.129080421	
979.539	0.27	0.580183	>0.05	0.383627	<0.01	0.468888	>0.05	978.5317	978.5385	−0.006897137	Titin
1503.572	0.27	0.520056	>0.05	0.362746	<0.01	0.447791	>0.05	1502.5647	1502.7365	−0.171821676	

## Discussion

In the present study, we reported time- and (region)-dependent changes in the expression of cardiac collagen, MMP/TIMP, and proteins of the cardiac sarcomere during the development of STZ-induced type 1 diabetes mellitus-associated diabetic cardiomyopathy. These alterations are associated with and likely contribute to changes in myocardial deformation behaviour as indicated by altered GLS and GCS.

Patients suffering from diabetic cardiomyopathy exhibit distinct cardiac structural and functional alterations. STZ application is a suitable method to induce experimental type 1 diabetes mellitus-associated diabetic cardiomyopathy characterized by enhanced cardiac inflammation and fibrosis^3,30–32^. There is increasing evidence that diastolic function is impaired already at early stages of diabetic cardiomyopathy, in the absence of prominent cardiac inflammation and fibrosis^4^.

However, the detection and mechanistic understanding of early cardiac dysfunction associated with pathological remodelling, has been a major challenge for several decades of research^33,34^. STE-based strain analysis provides an attractive tool for the assessment of global cardiac function and allows the detection of subtle changes in tissue deformation. As such, recent guidelines for diagnosis of heart failure include the assessment of GLS^9^. However, the link between myocardial deformation and underlying changes in structural proteins in the context of diabetic cardiomyopathy is still little explored. Only a few studies^5,24,35^ have determined global LV function via strain parameters in the context of experimental diabetic cardiomyopathy so far. Therefore, the aim of our study was to evaluate myocardial deformation in combination with the underlying changes in the expression of structural components during the pathogenesis of STZ-induced type 1 diabetes mellitus-associated diabetic cardiomyopathy.

Here, we observed an impairment of GLS and GLS rate after STZ application, which is consistent with findings in patients suffering from heart failure with preserved ejection fraction^13^ and diabetes mellitus^14^. Additionally performed regional peak strain analysis implicated that these changes in GLS, which were the most prominent at 9w, were probably caused by less myocardial contraction of the anterior and/or posterior base. Supporting the recently proposed hypothesis that a loss in longitudinal deformation behaviour is compensated by improved circumferential deformation behaviour^15^, we detected an enhancement in GCS and GCS rate in 6w and 12w STZ mice. In contrast, GCS and GCS rate of 9w STZ mice were reduced relative to 6w and 12w STZ mice, and this effect was paralleled by a lack of changes in segmental contraction. Interestingly, Shepherd and colleagues, who also used a STE-based approach, detected no change in GLS in 6w STZ mice, whereas GCS, global radial peak strain and subsequently strain rates were reduced^24^. In contrast to our study, in which we performed quantitative analysis on each of the 6 single segments, Shepherd and colleagues grouped the long- and short axis images only into 2 segments, which disables a direct comparison between the studies. However, we believe that by the grouping of segments, the added value of the regional analysis, which is actually the advantage of this method, goes lost. Therefore, we are not surprised that the authors only reported regional changes of the global radial peak strain, but not of the GLS. In addition to GLS and GCS, we also investigated DWS as a marker of diastolic function. Non-invasive assessment of LV wall stiffness in experimental models was first established by Takeda *et al*.^25^ who reported an inverse correlation of DWS with myocardial stiffness. Despite the lack of correlation in larger patient cohorts^26^, reduced DWS may be associated with a decreased diastolic function^27^, which has been confirmed by our findings.

As first stated by Greenbaum and colleagues in 1981^6^, the LV consists of longitudinal and circumferential fibres, with GLS being mainly determined by contraction of longitudinal fibres^8^. For diabetes mellitus, it is hypothesized that longitudinal fibres are more affected by extracellular matrix turnover^16^. In our study, we detected a reduction in GLS, which is mainly determined by longitudinal fibres. In parallel, collagen I deposition was increased at 9w STZ. Interestingly, we observed less collagen I at 12w post-STZ compared to 9w STZ, paralleled by lower Lox and LoxL2 levels, as markers for collagen cross-linking^36^. The reduction in collagen at 12w STZ *per se* is contradictory to already published studies, reporting higher collagen levels^3,23,31^. Only one study, investigating the effect of thioredoxin-interacting protein, reported less myocardial collagen at 16w STZ compared to controls^37^. But alterations in extracellular matrix, especially collagen, are not only determined by production and cross-linking, but also by degradation. Extracellular matrix degradation is predominantly regulated by MMPs, which are counteracted by TIMPs. Several studies have previously connected the dysregulation in MMPs/TIMPs and subsequent collagen deposition in STZ-induced type 1 diabetes mellitus-associated diabetic cardiomyopathy with an impairment of myocardial function^31,38^. With respect to our study, changes in cardiac MMP-8 and MMP-13 gene expression levels were observed after STZ application, suggesting a time-dependent modulation in collagen degradation.

Recently, we have demonstrated layer-specific changes in extracellular matrix proteins, which resulted in layer-specific changes of strain parameters in an experimental model of isolated subendocardial fibrosis^20^. Based on these findings, IMS was applied to further investigate global and regional-dependent changes in cardiac protein signature. In accordance to previous proteome analysis in experimental type 1^39^ and type 2 diabetes^40^, we detected alterations in intensity distribution of proteins, which are associated with cardiac energy metabolism and contractile function and play a crucial role in the progression of diabetic cardiomyopathy^41^. The higher distribution of α-actin, myosin light chain 3, ATP synthase, and titin in 6w STZ mice compared to control animals, might be a compensatory mechanism to counteract the reduced GLS and could hypothetically be reflected in the increased GCS. Similar observations were made in cardiac contractility modulation patients, where improved cardiac function was associated with an increased expression of contractile proteins, including myosin light chain and titin^42^. In contrast to that, the intensity distribution of all mentioned protein signatures was reduced in 9w STZ mice versus controls, which was paralleled by an increased collagen I/III ratio. These changes probably underlie the impaired GCS and GLS, indicative for reduced myocardial deformation behaviour. At 12w post-STZ, mice exhibited decreased myocardial α-actin, myosin light chain 3, and ATP synthase levels compared to controls. Nevertheless, titin was unaffected in 12w STZ mice, which might be an explanation for the lack of significant improvement in GCS compared to the respective controls, whereas GCS at 12w post-STZ was increased relative to 9w post-STZ.

### Future directions and limitations

Given the high sensitivity of STE-derived myocardial deformation data (global peak strain and global peak strain rate), this technique is recommended as a non-invasive diagnostic method for extensive clinical use^43,44^. Both data allow the early detection of myocardial dysfunction of different aetiologies and provide prognostic information. They are easy to acquire for a trained expert in echocardiography and helpful for therapeutic decision making and follow-up examinations. Despite these valuable advantages, clinical application of STE is still challenging^45^. Only standardization of measurements, continued training, and quality assurance ensure less measurement differences between various vendors/observers and allow an accurate interpretation. To date, STE-derived myocardial deformation data only in conjunction with other parameters, such as ejection fraction, are used for clinical decision making. The combination of STE with IMS allows time- and region-dependent alterations on molecular and functional outcome in the pathogenesis of STZ-induced type 1 diabetic cardiomyopathy. Especially, since it was not expected to see this specific cardiac phenotype found at 12w post STZ, including a decrease in collagen content, in comparison to the 9w and 6w post STZ time points. With respect to the existing literature, our findings are contradictory and were only confirmed by Myers *et al*.^37^. This discrepancy in findings might be explained by the STZ model. Even if the STZ-induced type 1 diabetes mellitus model is considered as the standard model for experimental type 1 diabetic cardiomyopathy, it should be taken into account that STZ is a chemical cytotoxic glucose analogue^46^. In this sense, the STZ-induced type 1 diabetes mellitus model can be regarded as a model of secondary (toxic) diabetes mellitus, which differs from human type 1 diabetes mellitus, which is an autoimmune disorder^46,47^. Furthermore, since no uniform protocol to induce type 1 diabetes mellitus in rodents exists^48,49^, this consequently results in different severities of experimental diabetic cardiomyopathy (e.g. rats display a more severe phenotype compared to mice), which ultimately leads to a poor comparability of experimental type 1 diabetes mellitus models in rodents. This highlights the need for comprehensive cardiac phenotyping in the context of experimental diabetic cardiomyopathy and supports the value of our study.

## Conclusions

This experimental study in an early stage model of type 1 diabetes mellitus-associated diabetic cardiomyopathy illustrates the strengths of two-dimensional STE to detect subtle, segmental changes in LV deformation important for the early diagnosis of diabetic cardiomyopathy and, in general, to assess ongoing cardiac remodelling processes. The combination of STE with IMS, allowing the respective assessment of region-dependent functionality and protein expression, is unique in its kind. We are the first to our knowledge combining both imaging techniques. The present study illustrates that during the development of STZ-induced type 1 diabetes mellitus-associated diabetic cardiomyopathy the expression of structural and contractile proteins shifts in a time- and region-dependent manner.

## Methods

### Animals

Type 1 diabetes mellitus was induced in eight-week-old male C57BL6/j mice (Charles Rivers, Sulzfeld, Germany) via intraperitoneal injection of STZ (50 mg/kg body weight; Sigma-Aldrich, Taufkirchen, Germany, n = 14) on 5 consecutive days^4^. In parallel, controls received the appropriate volume of 0.1 mol/L sodium citrate (n = 12). Blood glucose measurements using the Accu-Chek Aviva® (Roche Diabetes Care Deutschland GmbH, Mannheim, Germany) were performed one week before STZ application and afterwards once a w to confirm hyperglycemia after 4 hours (h) fasting. Mice were group housed under standard housing conditions (12 h light/dark cycle, 50–70% humidity, 19–21 °C) within a specific pathogen free facility with food and water *ad libitum*. Cages were enriched with houses, bedding material, and gnawing sticks. All investigations were performed in accordance with to the European legislation for animal welfare (Directive 2010/63/EU) and approved by the local ethics committee (Landesamt für Gesundheit und Soziales, Berlin, G0254/13).

### Echocardiography

Six, 9, and 12w after type 1 diabetes mellitus induction, echocardiographic measurements were performed using a Vevo 3100 Imaging System coupled to a 30-MHz linear-frequency transducer (FUJIFILM VisualSonics, Toronto, ON, Canada). According to our previously published standard operating protocol^20^, mice were anesthetized with isoflurane (Abbott Laboratories, Abbott Park, IL, USA) and fixed in a supine position. To obtain constant heart rates, isoflurane concentrations were reduced to 1–2%. Next, mice were depilated using hair-removal crème, and pre-warmed ultrasound gel (Parker Laboratories, Fairfield, NJ, USA) was placed on the chest. B- and M-Mode images were acquired in parasternal long axis and short axis view. A trained expert in small animal echocardiography performed STE analysis of the B-Mode images in a semiautomatic manner using the VevoStrain software package (FUJIFILM VisualSonics), as reported previously^20^. Determination of cardiac systolic function was performed in a semiautomatic manner from the respective B-mode images (long axis view) using the monoplane Simpson method of disks. To calculate LV dimensions and LV mass, the acquired M-Mode images of the parasternal long axis view were used. In detail, three independent M-Mode images and 3 cardiac cycles were analysed. To determine diastolic function, DWS was calculated as previously described^27^.

### Tissue preparation for molecular investigations

To examine gene and protein expression, an additional set of experiments was performed. To this end, type 1 diabetes mellitus was induced as mentioned above. At the respective time points (6w, 9w, and 12w after STZ application or control treatment), 5–6 mice/group were euthanized via cervical dislocation and the LV was excised, immediately snap frozen in liquid nitrogen and stored at −80 °C until further processing. To confirm characteristics of the echocardiographic set, blood glucose levels, body weight, LV weight and LV weight/body weight ratio derived from the second set of animals were depicted (Supplemental Fig. 3).

### RNA isolation and gene expression analyses from heart tissue

LV RNA was isolated using the TRIzol^®^ method, as described in detail elsewhere^50^. According to manufacturer’s protocol, the RNA pellet was purified with the RNeasy Mini Kit (Qiagen, Hilden, Germany) and subsequently transcribed into cDNA using the High Capacity Kit (life Technologies, Darmstadt, Germany). Real-time PCR was performed using the following gene expression assays (all provided by Applied Biosystems, Darmstadt, Germany): Col1a1 (Mm01302043_g1), Lox (Mm00495386_m1), LoxL-2 (Mm00804740_m1), MMP-8 (Mm00439509_m1), MMP-13 (Mm00439491_m1), and TIMP-1 (Mm00441818_m1). Gene expression levels were normalized towards CDKN1b (Mm00438167_g1) and expressed as the n-fold change to the respective control group, set as 1.

### Immunohistology

Frozen LV were embedded in Tissue-Tek (Sakura, Staufen, Germany) for cryosectioning and subsequent staining. As described previously^51^, specific antibodies directed against Collagen I (Chemicon, Limburg/Lahn, Germany) and Collagen III (Merck Millipore, Darmstadt, Germany) were used. Quantitative analysis of the positive area (%)/heart area (HA; mm^2^) was performed by digital image analysis on a Leica DM2000 light microscope (Leica Microsystems, Wetzlar, Germany) at 100× magnification.

### Left ventricular proteome analysis by MALDI-Imaging mass spectrometry

MALDI-IMS was performed as previously described^52^. In brief, formalin-fixed paraffin embedded (FFPE) LV sections of 6 µm were transferred onto Indium-Tin-Oxide slides (Bruker Daltonik, Bremen, Germany), dewaxed and passed through decreasing concentrations of ethanol^53^. Next, trypsin solution was applied onto the sections via an automated spraying device (ImagePrep; Bruker Daltonik) for 3 h incubation (37 °C; moist chamber). Finally, matrix solution (α-cyano-4-hydroxycinnamic acid) was applied using ImagePrep. For MALDI-IMS data acquisition, the positive ion reflector mode on an MALDI-TOF/MS (Autoflex III; Bruker Daltonik) was used. The corresponding detection range and raster width were set as 800–3500 Da and 100 µm, respectively. After MALDI-IMS experiments, tissue sections were stained with haematoxylin and eosin (H/E) to define the region of interest. As previously reported^53^, m/z values were identified via complementary protein identification using the “bottom-up”-nano liquid chromatography (Dionex Ultimate 3000, Thermo fischer) - MS/MS (ESI-QTOF; Bruker Daltonik) approach. Mass spectra were analysed using the mascot search engine (version 2.4, MatrixScience, UK) searching the UniProt database. To this end, the following parameters were set: (i) taxonomy: mus musculus (ii) proteolytic enzyme: trypsin, (iii) peptide tolerance: 10 ppm, (iv) maximum of accepted missed cleavages: 1, (v) peptide charge: 2+, 3+, 4+; (vi) variable modification: oxidation (M); (vii) MS/MS tolerance: 0.05 Da, and (viii) MOWSE score > 25. Identification of MALDI-IMS m/z values by using an LC-MS/MS reference list requires the accordance of more than one peptide (mass differences <0.9 Da) to subsequently correctly assign the corresponding protein^54^.

### Statistical analysis

All Data are expressed as mean ± SEM and statistical analysis was performed using the GraphPad Prism 8 Software (GraphPad Software, La Jolla, CA, USA). Differences between the groups were assessed using the One-way ANOVA (Fisher´s LSD post hoc test) or Kruskal-Wallis Test (Dunn´s post hoc test) and considered significant at a value of p < 0.05. For IMS-proteome data, statistical analysis was performed using the SCiLS Lab software (Version 2015b, SCiLS GmbH, Bremen, Germany), as described before^52^. To define common molecular features among the sample sets, unsupervised multivariate principal component analysis for mass spectra was applied for each time point separately. The mesoderm was defined at the corresponding H/E staining as region of interest from STZ as well as controls. Additionally, principal component analysis was performed in the STZ group at the different time points. A receiver operating characteristic (ROC) analysis was performed pairwise (STZ vs control) to identify discriminating properties (peptides) in the mesoderm of LV tissue specimens at 6w, 9w, and 12w after type 1 diabetes mellitus induction. For those peaks with a ROC score of AUC > 0.6 or <0.4, a univariate hypothesis test (Wilcoxon rank sum test) was used to ensure the statistical significance of m/z values. Results with significant differences (p-value ≤ 0.01) in the Wilcoxon test were assumed to be potential markers.

## Supplementary information

Supplementary Information.

## Data Availability

The datasets used and/or analysed during the current study are available from the corresponding author on reasonable request.
