# Supplementary material for: Speckle-tracking echocardiography combined with imaging mass spectrometry assesses region-dependent alterations
Source: Sci Rep. 2020 Feb 27;10:3629. doi: 10.1038/s41598-020-60594-2 (PMC7046677; doi:10.1038/s41598-020-60594-2)
Supplement: Supplementary file 1 — Supplementary Information. [file 41598_2020_60594_MOESM1_ESM.docx]

# SUPPLEMENTAL MATERIAL

# Speckle-tracking echocardiography combined with imaging mass spectrometry assesses region-dependent alterations

Kathleen Pappritz^1,2#^, PhD; Jana Grune^2,3,4#^, PhD; Oliver Klein^1,2^, PhD; Niklas Hegemann^2,3^, M.Sc. ; Fengquan Dong^1^, MD ; Muhammad El-Shafeey^1,5^, PhD; Jie Lin^1^, MD ; Wolfgang M. Kuebler^2,3^, MD; Ulrich Kintscher^2,4^, MD; Carsten Tschöpe^1,2,6^, MD; Sophie Van Linthout^1,2,6^, PhD

^#^ equal contribution

^1^ Berlin Institute of Health Center for Regenerative Therapies & Berlin-Brandenburg Center for Regenerative Therapies (BCRT), Charité – Universitätsmedizin Berlin, Campus Virchow Klinikum (CVK), Berlin, Germany

^2^German Center for Cardiovascular Research (DZHK), Partner site Berlin, Berlin, Germany

^3^Institute of Physiology, Charité – Universitätsmedizin Berlin, Charité Campus Mitte (CCM), Berlin, Germany

^4^Center for Cardiovascular Research (CCR), Institute of Pharmacology, Charité – Universitätsmedizin Berlin, Berlin, Germany

^5^ Medical Biotechnology Research Department, Genetic Engineering and Biotechnology Research Institute (GEBRI), City of Scientific Research and Technological Applications, Alexandria, Egypt

^6^ Department of Cardiology, Charité – Universitätsmedizin Berlin, Campus Virchow Klinikum (CVK), Berlin, Germany

# * Correspondence

Sophie Van Linthout, PhD

Berlin Institute of Health Center for Regenerative Therapies &

Berlin-Brandenburg Center for Regenerative Therapies (BCRT)

Charité – Universitätsmedizin Berlin

Campus Virchow Klinikum (CVK)

Föhrer Strasse 15

13353 Berlin

e-mail: sophie.van-linthout@charite.de

Phone: +49-(0)30-450539486

Fax: +49-(0)30-450539409

# Supplemental Figure 1.


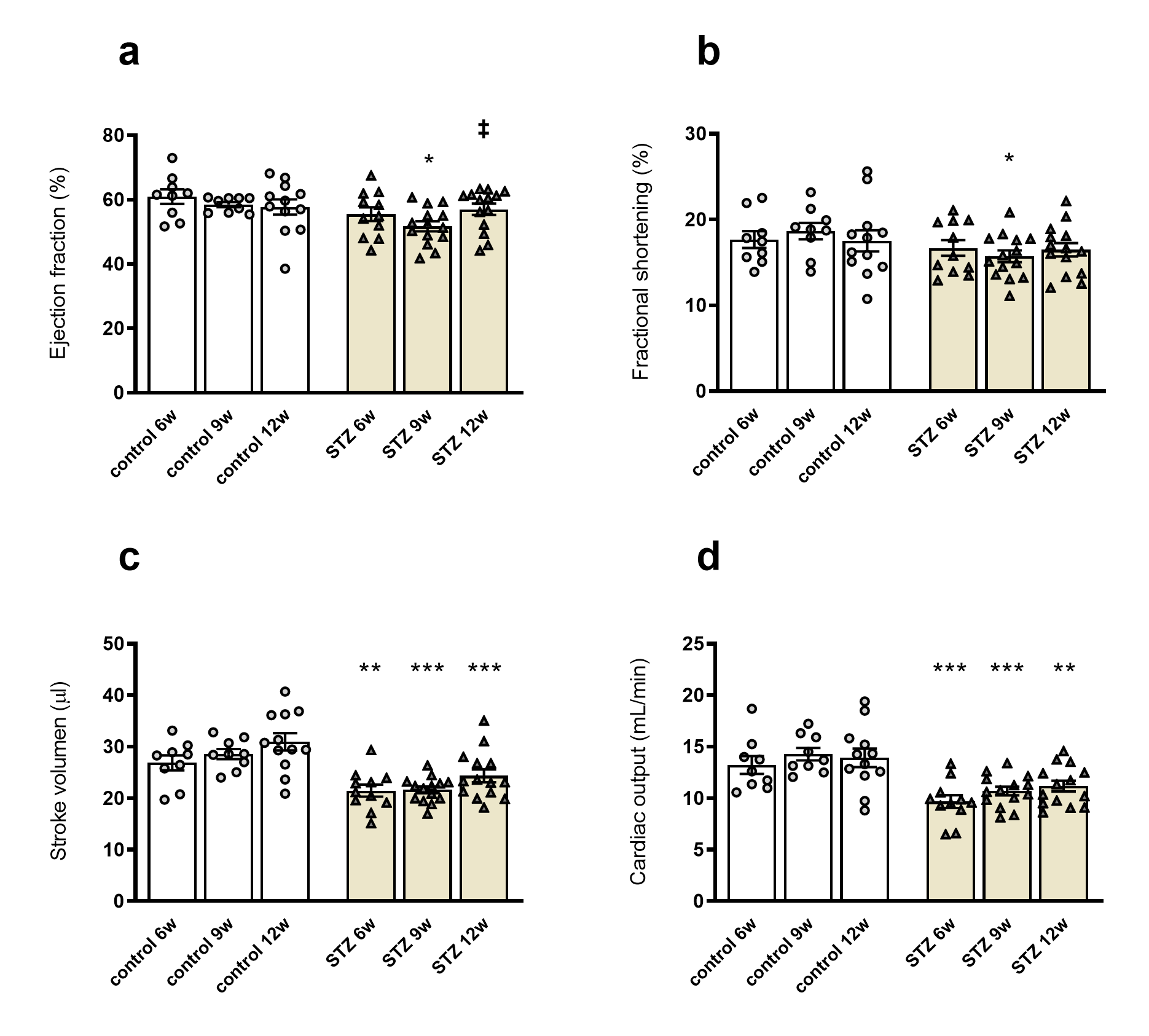


**Supplemental Fig. 1. STZ-induced type 1 diabetes mellitus modulates systolic function.** Investigation of LV ejection fraction (**a**), fractional shortening (**b**), stroke volume (**c**), and cardiac output (**d**) in the pathogenesis of STZ-induced diabetes mellitus-associated cardiomyopathy. Bar graphs represent the mean±SEM. Data were analysed with One-way ANOVA or Kruskal-Wallis test (*p<0.05; **p<0.01, ***p<0.001, ***p<0.0001 versus corresponding control; †p<0.05, ††p<0.01, †††p<0.001, ††††p<0.0001 versus the 6w STZ; ‡p<0.05, ‡‡p<0.01, ‡‡‡p<0.001, ‡‡‡‡p<0.0001 versus the 9w STZ; n=12/controls and n=14/STZ).

# Supplemental Figure 2.


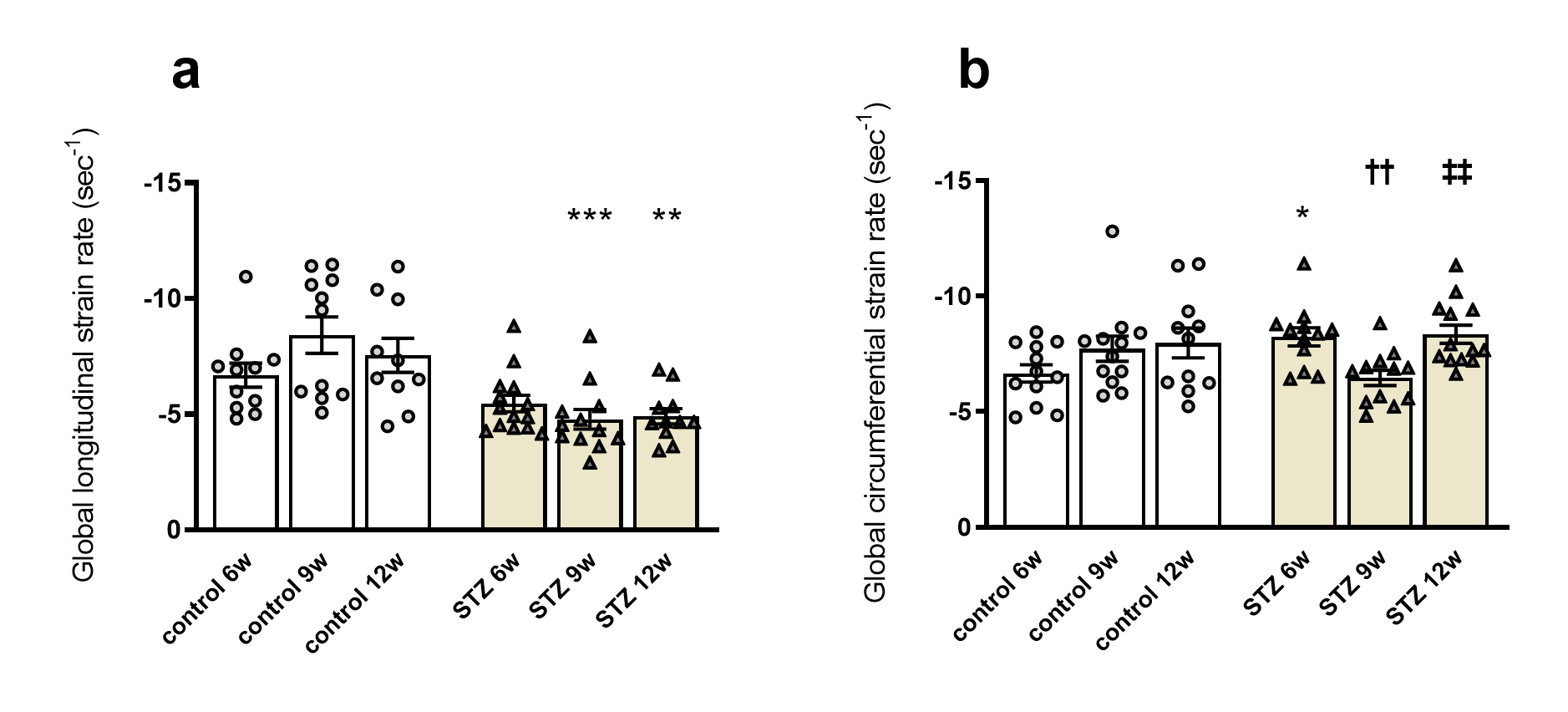


**Supplemental Fig. 2. STZ-induced type 1 diabetes mellitus reduces global strain rate.** Determination of the global longitudinal strain rate (**a**) and global circumferential strain rate (**b**) via two-dimensional speckle-tracking echocardiography (STE). Bar graphs represent the mean±SEM. Data were analysed with One-way ANOVA or Kruskal-Wallis test (*p<0.05; **p<0.01, ***p<0.001, ***p<0.0001 versus corresponding control; †p<0.05, ††p<0.01, †††p<0.001, ††††p<0.0001 versus the 6w STZ; ‡p<0.05, ‡‡p<0.01, ‡‡‡p<0.001, ‡‡‡‡p<0.0001 versus the 9w STZ; n=12/controls and n=14/STZ).

# Supplemental Figure 3.


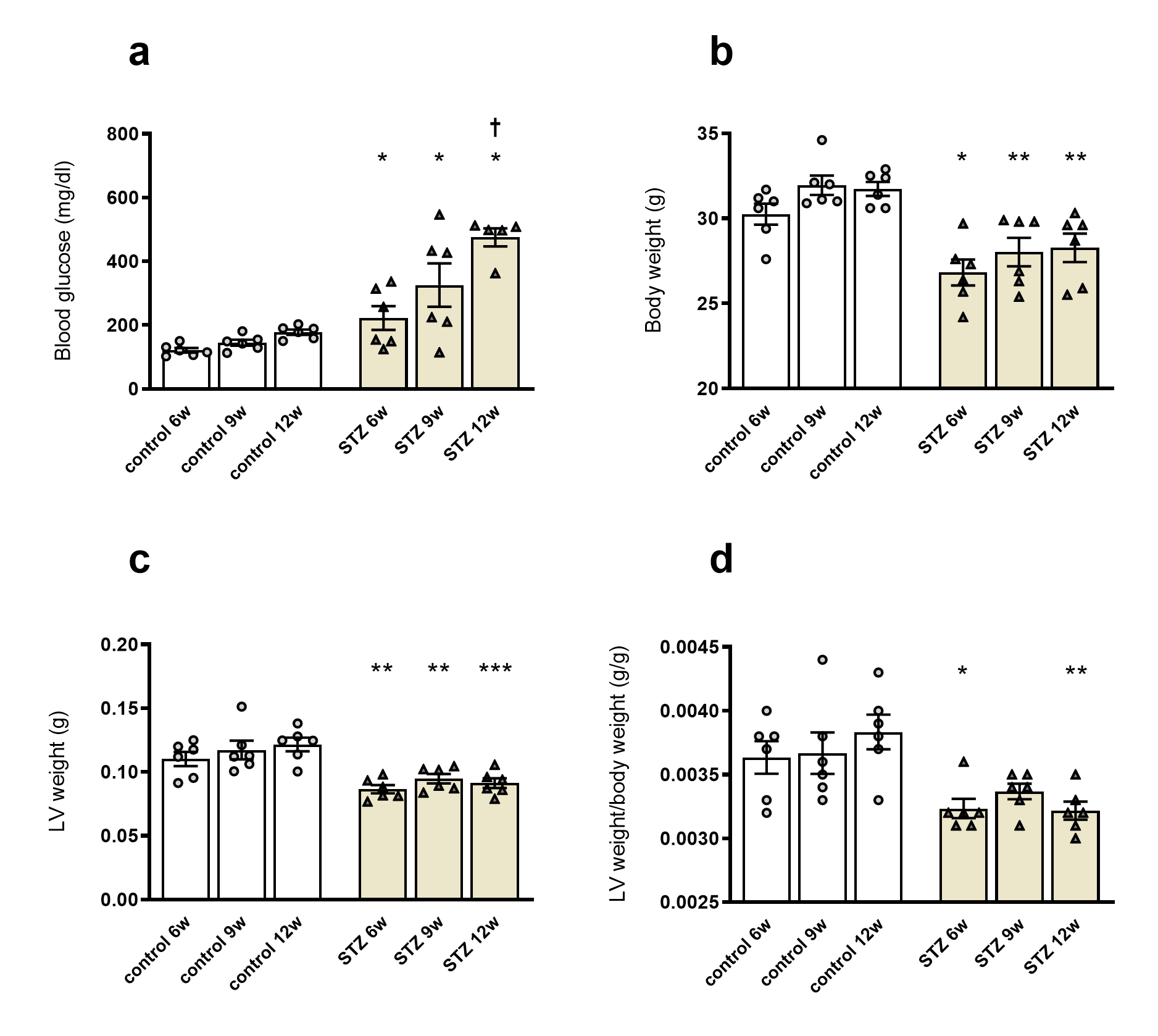


**Supplemental Fig. 3. STZ-induced type 1 diabetes mellitus is associated with impaired morphological parameters. a**) Blood glucose levels (mg/dl) in control mice and 6w, 9w, and 12w after STZ application derived from the second set of animals. Besides increased blood glucose, mice suffering from type 1 diabetes displayed lower body weight (g) over time (**b**). As morphological parameters, LV weight (g; **c**) and LV weight/body weight (g/g; **d**) are depicted. Bar graphs represent the mean±SEM. Data were analysed with One-way ANOVA or Kruskal-Wallis test (*p<0.05; **p<0.01, ***p<0.001, ***p<0.0001 versus corresponding control; ^†^p<0.05, ^††^p<0.01, ^†††^p<0.001, ^††††^p<0.0001 versus the 6w STZ; ^‡^p<0.05, ^‡‡^p<0.01, ^‡‡‡^p<0.001, ^‡‡‡‡^p<0.0001 versus the 9w STZ; n=5-6/controls and n=5-6/STZ).
